# Changes in paleo-groundwater levels revealed by water wells and their relationship with climate variations in imperial Southern China

**DOI:** 10.1371/journal.pone.0292662

**Published:** 2023-10-25

**Authors:** Chenyao Jiang, Harry F. Lee, Xin Jia, Xinggong Kong

**Affiliations:** 1 Department of Geography and Resource Management, The Chinese University of Hong Kong, Hong Kong, SAR, China; 2 School of Geography, Nanjing Normal University, Nanjing, China; 3 Jiangsu Center for Collaborative Innovation in Geographical Information Resource Development and Application, Nanjing, China; 4 Key Laboratory of Virtual Geographic Environment (Nanjing Normal University), Ministry of Education, Nanjing, China; 5 Insitute of Environmental Archaeology, Nanjing Normal University, Nanjing, China; Alagappa University, VIET NAM

## Abstract

Based on records of the bottom elevations of 511 ancient water wells from published archaeological reports, we reconstructed the paleo-groundwater levels (PGWL) in urban areas of Chengdu, Changsha, Nanjing, Suzhou, Suqian, Yancheng, Fuzhou, and Guangzhou cities in southern China. Our PGWL reconstruction shows that PGWL varied in two patterns. In the inland monsoon region (Chengdu and Changsha), there was a low PGWL in Jin (AD 266–420) and South Song (AD 1127–1279), and a high PGWL in Tang (AD 618–907) and Ming (AD 1368–1644). In the coastal region (Yancheng, Fuzhou, and Guangzhou), there was a low PGWL in Jin (AD 266–420) and Ming (AD 1368–1644) but a high PGWL in Tang (AD 618–907) and Song (AD 960–1279). Via cross-wavelet transform and wavelet transform coherence analyses, we found that monsoon and temperature significantly drove the PGWL fluctuations at the inter-centennial scale. East Asian Summer Monsoon-induced precipitation has continuously affected cities in the inland monsoon area represented by Chengdu and Changsha over the past 2,500 years. It has also intermittently affected Nanjing and Suzhou when EASM intensified. In parallel, temperature influenced the PGWL in coastal cities such as Yancheng, Fuzhou, and Guangzhou via the changes in the sea level. Also, the temperature affected the PGWL in relatively inland cities during climatic anomalies such as the Medieval Warm Period and Little Ice Age. This study demonstrates the value of archaeological records in learning how climatic factors influence the PGWL variation and its mechanism.

## Introduction

Water resources are essential for human survival and sustainable social development [1, 2]. Groundwater has been a primary water source for human needs since prehistoric times [3]. As transported water is no longer sufficient to meet the needs of human settlements, residents have developed methods of procuring and storing water to ensure a stable water supply [4]. The most traditional and straightforward method of drawing groundwater is by using water wells. In some Mediterranean countries and Near East regions with low surface water availability, the construction and use of wells can date back to the early Bronze Age [5–8] or even the Neolithic era [9, 10]. In China, the earliest well was a wooden one built around 6174–5921 cal. BP in the Hemudu archaeological site in Zhejiang [11], while wells were increasingly constructed after 5000 BP [3].

Water wells are built by excavating the ground and accessing groundwater in aquifers through digging, driving, boring, or drilling [3]. The preservation of ancient water wells provides direct indicators of paleo-groundwater level (PGWL) [12]. In some coastal regions, well bottoms have also been recognized as related to sea level [13]. Historical well studies suggest that well bottoms are well correlated with PGWL. For example, PGWL is 30 cm higher than well bottoms in Caesarea Maritima, Israel [14], and 60 cm above well bottoms on the Northern coast of Israel [4, 15]. In comparison, it is approximately 80–90 cm higher than well bottoms in the lower Yellow River region [16]. While the height difference between PGWL and well bottoms varies across regions, this difference has remained temporally consistent, making the well bottom a reliable indicator of PGWL. Indeed, the variation of well bottoms was employed to indicate sea-level fluctuation in Yancheng, China, between East Han (c. AD25–220) and Ming (c. AD1368–1644) [17].

Several studies have examined the factors that influence variations in groundwater levels, including natural factors such as climate and human activities [18–21]. Regarding climate factors, previous research has shown that precipitation and evapotranspiration play a role in groundwater level fluctuations [22]. Groundwater levels are also strongly correlated with regional temperature, which is linearly related to evapotranspiration [23, 24]. Additionally, the temperature can affect coastal water tables by inducing sea-level fluctuations [13]. Human activities, such as building dams [25] and using groundwater for irrigation and daily life [26], also impact the groundwater supply. However, there are still unanswered questions, such as: (1) How does groundwater respond to changes in precipitation or temperature [27, 28]? (2) Due to the low temporal resolution of sediment samples used in standard dating methods (e.g., cosmogenic nuclides, optically stimulated luminescence, radiocarbon, etc.), most studies on past groundwater levels only cover prehistoric periods. Furthermore, changes in paleo-water levels during the imperial period, which were closely related to the rapid development of human societies, have rarely been examined. Investigating historical variations in groundwater resources and their influencing factors is necessary for the long-term sustainability of agricultural production and regional development.

Nurtured by the East Asian Summer Monsoon (EASM), many well-known ancient civilizations originated in the Yangtze River Basin, such as the ancient Shu civilization (e.g., Baodun Culture, Sanxingdui Culture, Jinsha Culture, etc.), Qujialing-Shijiahe Culture, and Liangzhu Culture [29, 30]. Numerous water wells have been excavated in this area. We collected 511 ancient water well records from excavation reports from eight Chinese cities in Southern China. We established a 2,500-year time series of well depth variation in each city based on specific structural features of wells of each age, accumulation layers, and construction materials. By comparing the variation of well depth, we reconstructed the variation of PGWL. Furthermore, we examined the primary factors that drove the variation of PGWL in Southern China in the past.

### Study area

We selected South China as our study area due to the abundance of well-preserved wells that were built between the Spring and Autumn period (722–479 BC) and the Qing dynasty (AD 1644–1911), particularly in the Yangtze River Basin. Theinhabitants in the Yangtze River basin began using wells around 1,000 years before the Yellow River basin because the aquifer in the Yangtze River basin was sufficiently high [3].

**Fig 1:**
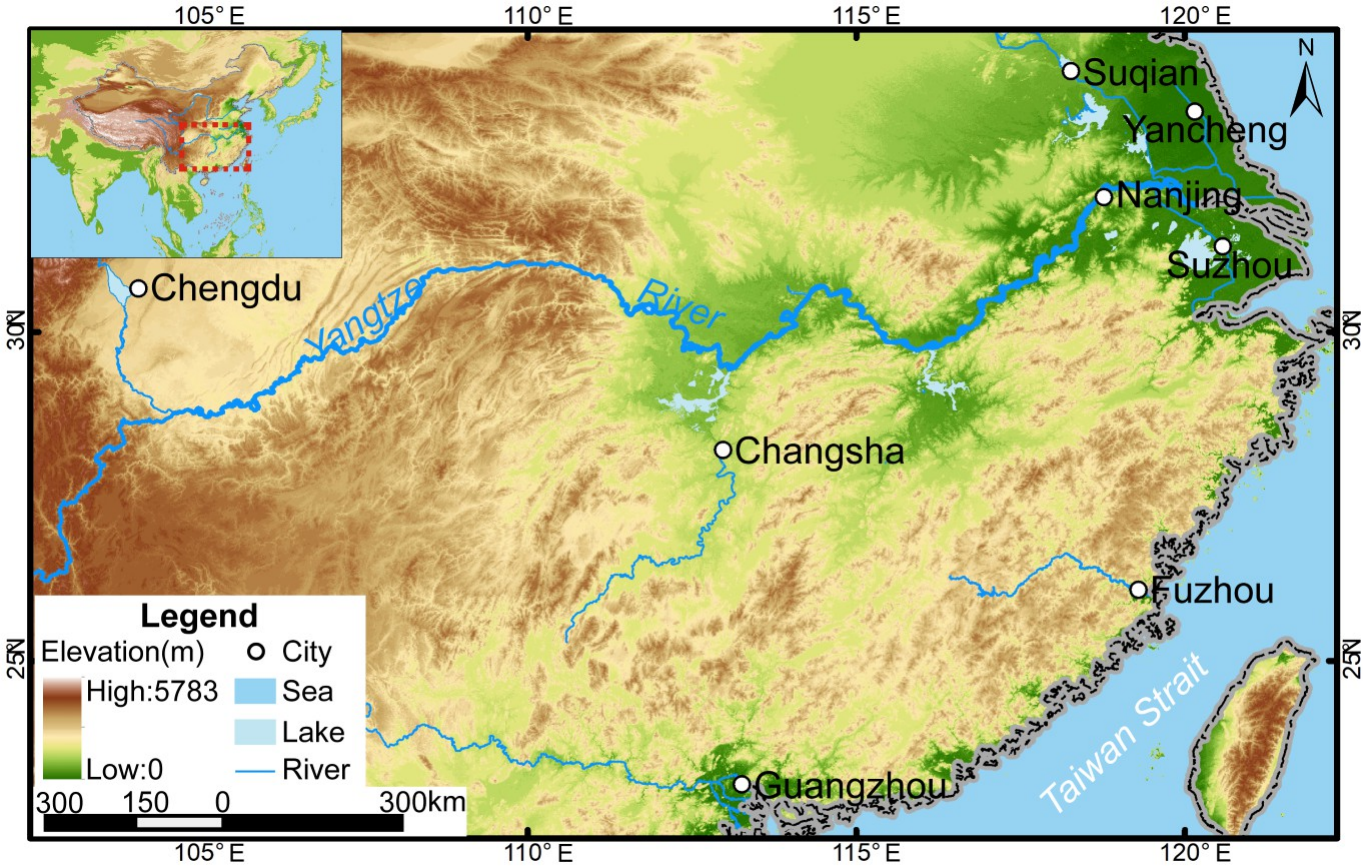
The location of the eight Southern China cities covered in this study. Maps were generated by ArcGIS 10.6 using open-source data. Source: DEM Data: Jarvis A, Reuter HI, Nelson A, Guevara E. Hole-filled seamless SRTM data V4. International Centre for Tropical Agriculture (CIAT); 2008. Available: https://srtm.csi.cgiar.org; Data of rivers: Berman L. China River Basins. 2011. Available: http://worldmap.harvard.edu/data/geonode:River_basin_num2; Data of lakes: ACASIAN. Major Lakes. Harvard Dataverse, V1; 2020. Available: https://doi.org/10.7910/DVN/76SIWT; Data of national boundary: OCHA Regional Office for Asia and the Pacific (ROAP). China—Subnational Administrative Boundaries. 2020. Available: https://data.humdata.org/dataset/cod-ab-chn?

We chose eight cities in southern China to collect water well records based on three criteria (Fig 1). First, we chose cities instead of counties because cities have more ancient water wells due to their larger population. Second, we excluded cities with rugged and undulating terrain as such features can obscure the effect of climate on groundwater level changes (e.g., Chongqing). Instead, we chose cities in relatively flat areas (e.g., Chengdu) because the groundwater is in a steady-state flow condition [4]. Finally, the chosen cities should have sufficient ancient water wells. For example, 171 ancient wells from the West Zhou dynasty (~1000 BC) were discovered at the Chenghu site in Suzhou City. Based on these criteria and the availability of archaeological records, we selected eight cities in our study area.

## Methods and materials

### Data

**Fig 2:**
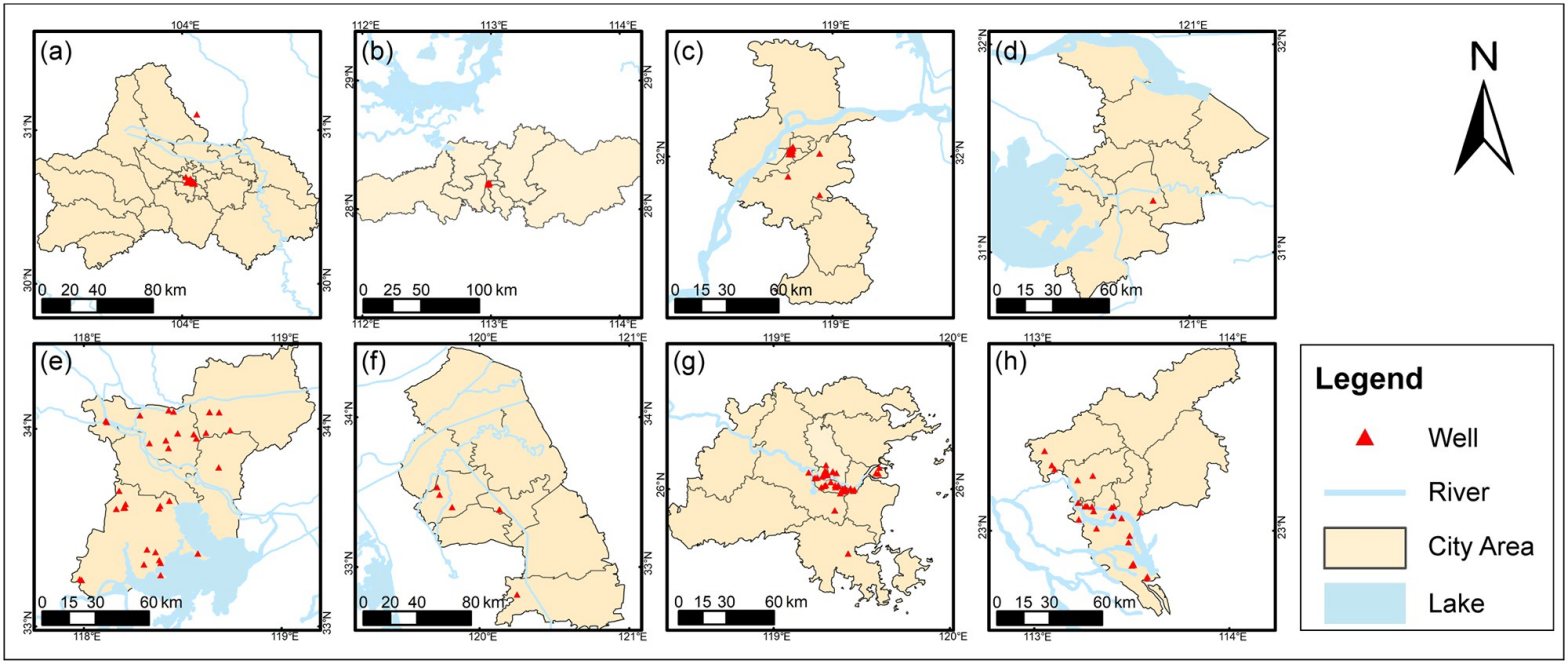
The distribution of ancient wells in the eight Southern China cities covered in this study. (a) Chengdu; (b) Changsha; (c) Nanjing; (d) Suzhou; (e) Suqian; (f) Yancheng; (g) Fuzhou; and (h) Guangzhou. Maps were generated by ArcGIS 10.6 using open-source data. Source: Data of rivers: Berman L. China River Basins. 2011. Available: http://worldmap.harvard.edu/data/geonode:River_basin_num2; Data of lakes: ACASIAN. Major Lakes. Harvard Dataverse, V1; 2020. Available: https://doi.org/10.7910/DVN/76SIWT; Data of administrative divisions: China Data Lab. China County Map with 2000–2010 Population Census Data. Harvard Dataverse, V1; 2020. Available: https://doi.org/10.7910/DVN/VKGEBX.

We obtained records of 511 ancient wells in Southern China, which contain the exact depth and building age of the wells (see Fig 2). These wells are documented in 53 excavation reports and have been meticulously examined to confirm their age. Various methods were used to determine the well ages, including identifying a specific feature of the well [31, 32], accumulation layers around the wells [33], dating of construction materials [16, 34], inscriptions on well curbs, and historical documents [33].

**Fig 3:**
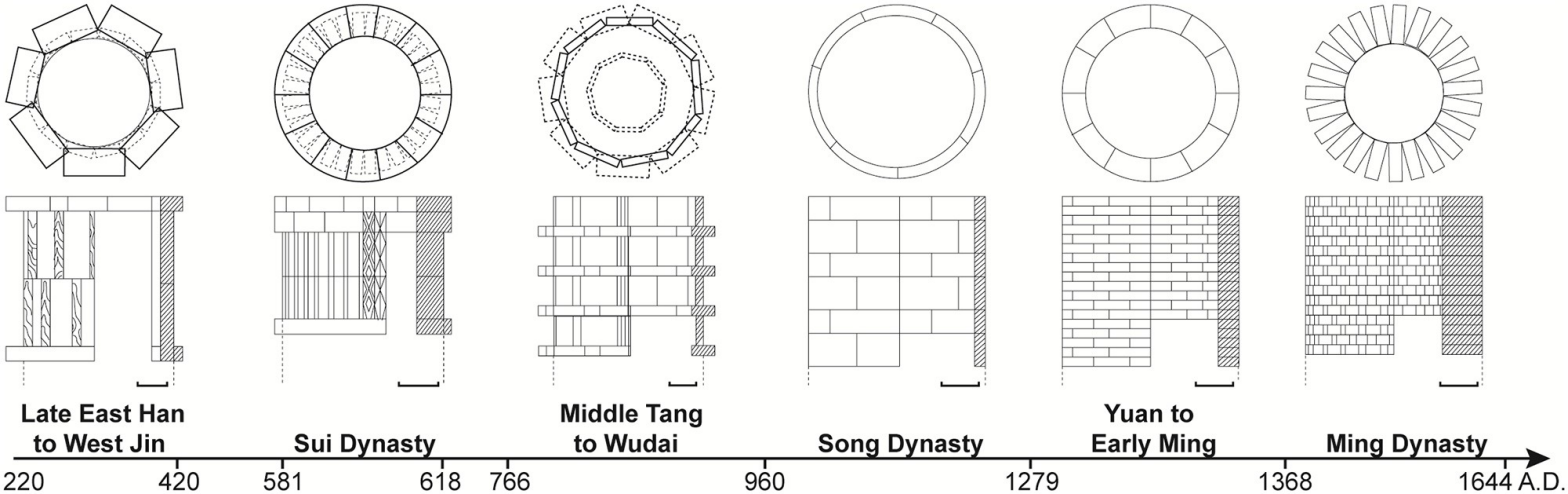
The evolution of the well morphology in the Jiangjun Road site, Yancheng city, based on Yu [35] (unit: 20 cm).

The most common dating methods in archaeological excavations are typological or cultural layer dating methods. By identifying the morphological characteristics of wells and comparing wells from different archaeological sites, the evolution of the well’s morphology can be revealed, helping to determine the relative age of each well. For example, 16 wells discovered along Middle Jianjun Road in Yancheng were classified into five epochs, and the shape characteristics of the wells in each epoch are summarized in Fig 3 [35].

Additionally, the construction date of wells can be determined by examining the accumulation layers both inside and outside the wells. The precision of these dating methods reaches the dynasty level and can even determine the phase of the dynasty (such as the Middle Tang dynasty). Radiocarbon dating is perhaps the most precise dating method. For instance, based on the C^14^ dating of the well wood, it was concluded that the oldest well in China was built in 5710 ± 125 BP [34]. However, this method is only applicable when construction materials or basal fills that can be dated by C^14^ (such as wooden side walls and bone remains) are available.

In parallel, inscriptions on some well curbs directly indicate the digging date of the well. For example, well No.1 in Xiaobaihua Alley in Nanjing is carved with the texts “*built by YongJian Zhao*, *during the Chunxi Bingwu years*,” indicating that it was built in the Chunxi year (AD 1186) of South Song (AD 1127–1279).

### Methods

#### Reconstruction of PGWL

Based on archaeological excavation data and related research on well archaeology, we have formulated the following hypothesis:

(1) The bottom elevation of a well indicates the long-term average height of the paleo-groundwater levels (PGWL). This is because the groundwater table in a well is usually higher than the bottom elevation. The common local water extraction containers determine this height difference to obtain water from wells [14] easily. Archaeological findings, such as jars in Israel and clay pots in eastern China [14, 36], show that the height of these containers buried at the bottom of wells is closely related to the long-term stable water level indicated by water rust on the well walls. Since water rust is not commonly found, we use the well bottom elevation to indicate the PGWL. This relationship can be expressed as follows:
$$Well\;bottom=H_{site}-H_{wellhead}-D$$
Wellbottom+ΔH=PGWL
  where *H*_*site*_ denotes the elevation of the site, *H*_*wellhead*_ is the height of the wellhead from the surface, *D* is the well depth, and the height of the local water extraction containers determines the value of Δ*H*. All these data are obtained from excavation reports and measured by traditional archaeological measurements [37]. As the water extraction method in the same area does not change much [3, 14], we assume that the difference between PGWL and the well bottom elevation does not change with time in the same region. Therefore, we use the elevation of well bottoms to indicate PGWL, and other studies of ancient wells also utilize this method [17, 38].
(2) The groundwater level is generally parallel to the ground surface in flat terrain areas. Ancient wells typically draw water from the phreatic water level, which undulates roughly in line with but flatter than the topography [16, 22, 23]. While the depth of the groundwater table is affected by elevation, we assume parallelity between ground and phreatic groundwater levels in small, flat areas. To control the effect of elevation differences, we obtain ancient well data from other nearby sites within the urban area of the city and calculate their mean values to represent the average groundwater level in the city. Moreover, since well bottoms are aligned with stable groundwater levels in the long run, we should consider the influence of long-term climate change rather than the seasonal regulation and storage effect of rivers and lakes on long-term groundwater levels.

Based on the above assumptions, we reconstructed the time series of PGWL in various cities using the bottom elevation of ancient wells, with dynasty as the temporal unit of the time series. Our data and reconstructions are provided in S1 File. When using dynasty as the temporal unit, the temporal resolution is 138±76 years (the maximum dating error does not exceed one dynasty, while the minimum error limit is within one year). Since the long-term PGWL variation is the focus of this study, such a temporal resolution is deemed acceptable.

Some regions have missing data on ancient wells in one or a few dynasties due to regional variations in the availability of archaeological materials. The most typical example is Suzhou, where most ancient wells were built during the Tang (AD 618–907) and Song (AD 960–1279), with no data on wells built afterward. Because the dynastic water well records in some cities are incomplete, we used the multiple imputation method to estimate the average well-bottom elevation for the dynasties with missing data. We selected the middle time point of each dynasty as the independent variable and imputed the mean PGWL of each dynasty using the Fully Conditional Specification method. We ran this process for 50 iterations and took their averages as the final results. We implemented this process using IBM SPSS Statistics 26. The calculated dynastic means of the PGWL of each city were employed in subsequent analyses.

#### Cross-wavelet transform and wavelet transform coherence methods

In addition to directly comparing changes in PGWL and climate fluctuations over the past 2,500 years, we employed cross-wavelet transform (XWT) and wavelet transform coherence (WTC) methods to examine the relationship between climate change and PGWL fluctuations. XWT uncovers regions where two time series have high common power. Grinsted et al. [39] defined it as:

$$W_{n}^{XY}\left(s\right)=W_{n}^{X}\left(s\right)W_{n}^{Y*}\left(s\right)$$

Where n is the time index and s is the scale; Wx and Wy are the XWT of two time series xn and yn; Wxy is cross wavelet power; * denotes complex conjugation;

WTC is defined by Torrence and Compo [40] as a tool to identify the Fourier squared coherence of the frequency bands of two time series covariance and find the regions where the two time series co-vary in the time-frequency space. According to Grinsted et al. [39], the WTC squared is:

$$R^{2}\left(s,t\right)=\frac{{\left|S\left(s^{-1}W_{XY}\left(s,t\right)\right)\right|}^{2}}{S\left(s^{-1}{\left|W_{X}\left(s,t\right)\right|}^{2}\right)\cdot S\left(s^{-1}{\left|W_{Y}\left(s,t\right)\right|}^{2}\right)}$$

Where S is a smoothing operator. Besides, the statistical significance level of WTC was determined by the Monte Carlo method. In this study, Morlet wavelets are used to decompose signals. We investigated the synchronicity of PGWL with temperature and precipitation variation over a specific periodicity using wavelet coherence. Crossed wavelets, on the other hand, are used to identify areas where the intensity of this periodicity is consistent to understand better the role and impact of climate on PGWL at long-term scales.

## Results

**Fig 4:**
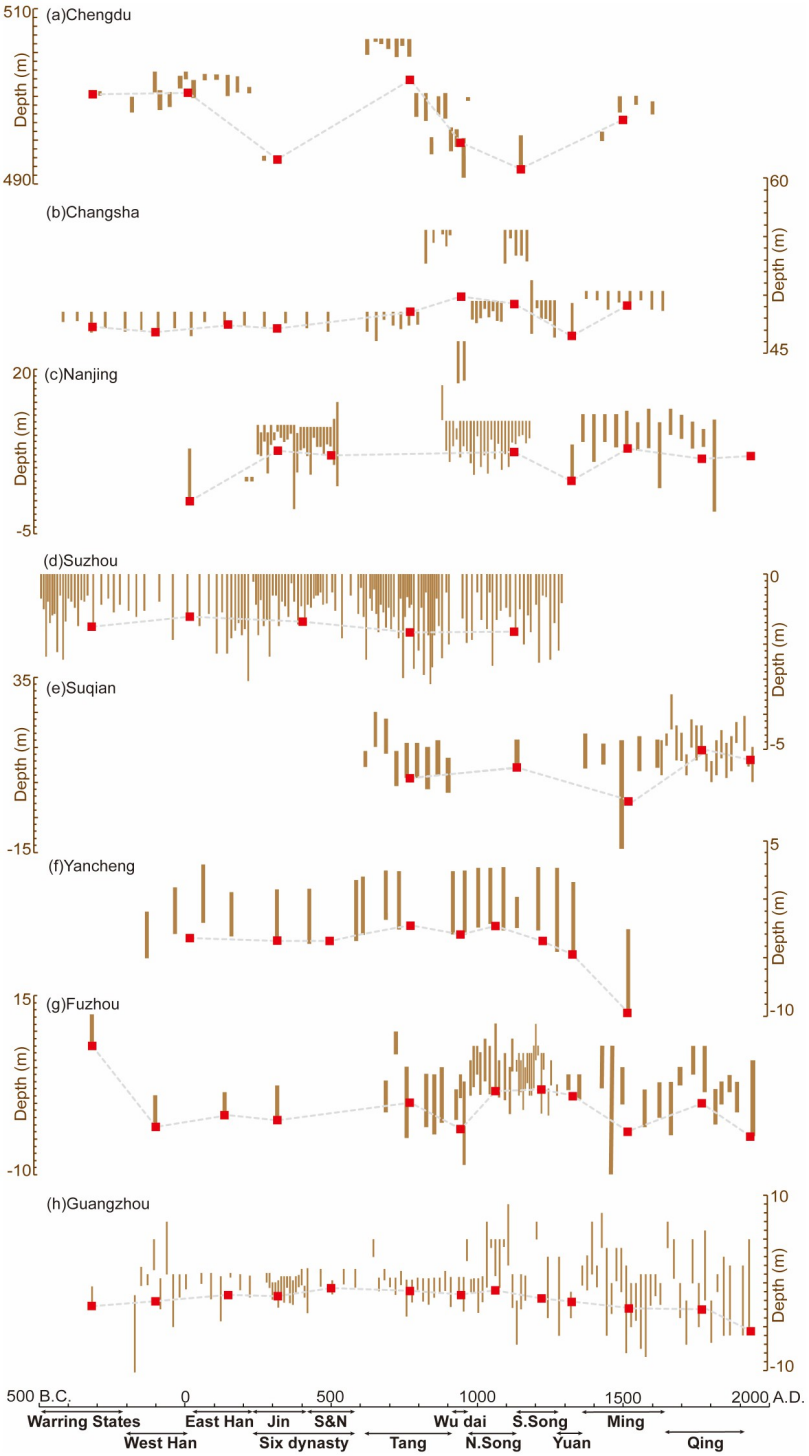
Height changes of the mouth and the bottom of ancient water wells (unit: m). The short bars indicate the relative depth of the water wells. The time coordinate corresponding to each water well only indicates in which dynasty (not the exact calendar year) the well was built. The red square denotes the dynastic means of the groundwater table in each city.

**Fig 5:**
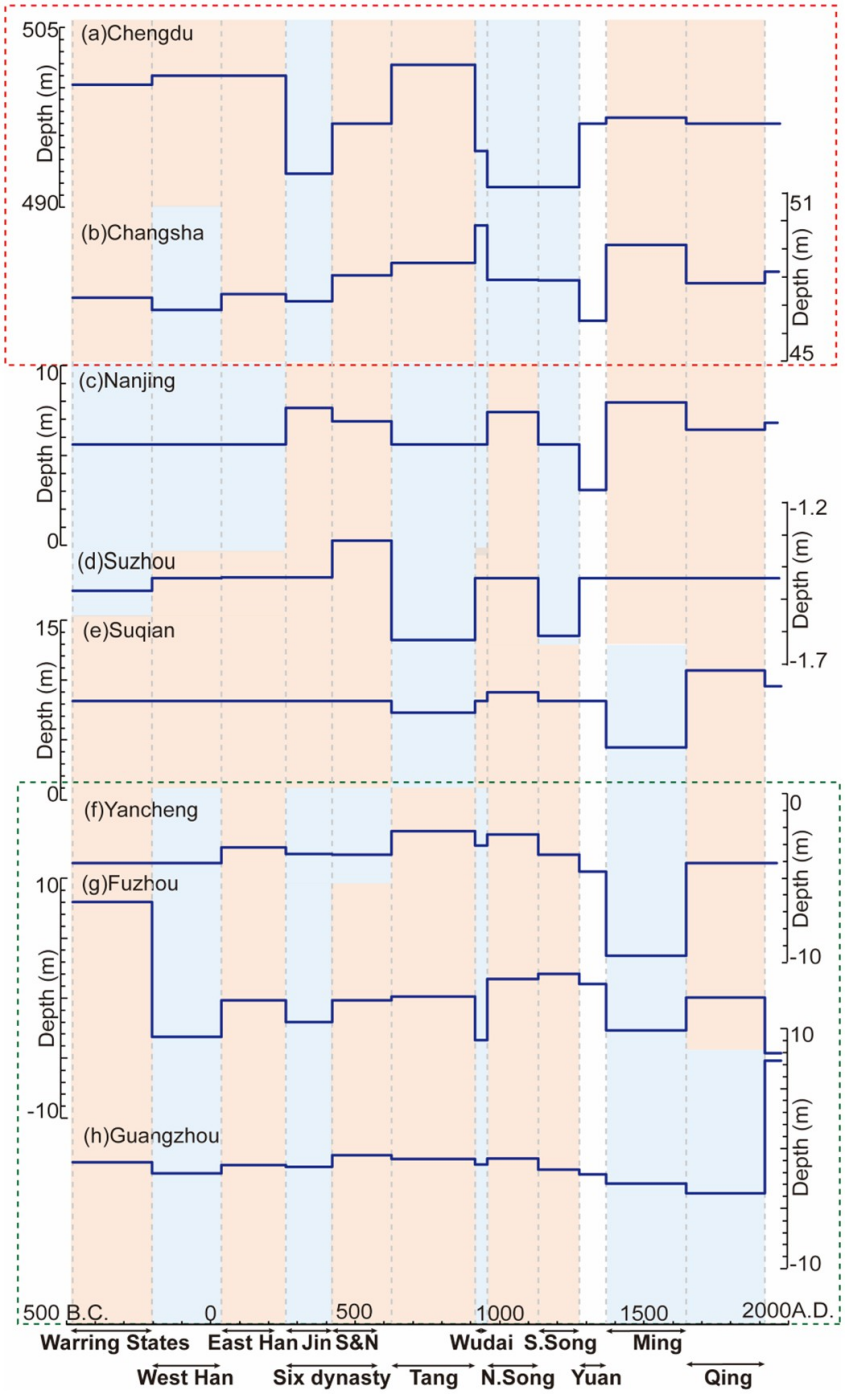
PGWL in Southern China cities. The dark blue line represents the dynastic means of the PGWL in each city. The orange shadow represents the periods with a high PGWL, while the blue shadow represents the periods with a low PGWL. The red dotted box indicates the PGWL in the inland monsoon region, while the green dotted box indicates the PGWL in the coastal region.

Eight cities were selected to reconstruct variations in PGWL, including Chengdu, Changsha, Nanjing, Suzhou, Suqian, Yancheng, Fuzhou, and Guangzhou (Fig 4). We calculated the average groundwater table of each dynasty in each city (Fig 5). Since 2500 BP, the overall mean PGWL is 496.74 m a.s.l. in Chengdu, 48.26 m a.s.l. in Changsha, 6.14 m a.s.l. in Nanjing, –1.46 m a.s.l. in Suzhou, 8.22 m a.s.l. in Suqian, –4.02 m a.s.l. in Yancheng, –0.27 m a.s.l. in Fuzhou, and –1.00 m a.s.l. in Guangzhou, respectively. Based on the overall mean PGWL of each city, we further categorized the dynastic mean PGWL in each city into two categories: (1) above or (2) below the mean PGWL. This categorization was used to help identify relatively high and low PGWL periods in each city.

### Variations of PGWL over the past 2500 years

Fig 5 shows that PGWL varies in two distinctive patterns: one similar to Chengdu and Changsha (inland monsoon region) and the other identical to Yancheng, Fuzhou, and Guangzhou (coastal region). The relatively high PGWL occurred in inland cities (i.e., Chengdu and Changsha) during Tang (AD 618–907) and Ming (AD 1368–1644), including 501.88 m a.s.l. and 497.48 m a.s.l. in Chengdu, and 48.511 m a.s.l. and 49.138 m a.s.l. in Changsha. In contrast, the low PGWL occurred during Jin (AD 266–420) and South Song (AD 1127–1279), with the PGWL values of 492.80 and 491.70 m a.s.l. in Chengdu and 47.13 and 47.87 m a.s.l. in Changsha. This pattern is aligned with some historical records of inrush and drought caused by changes in groundwater levels. For instance, *Chronology of Major Natural Disasters and Anomalies in Ancient China* [41] states that there were 15 records of nationwide mega-droughts during Jin (AD 266–420), compared with five in Sichuan and Hunan during Song (AD 960–1279). In comparison, these two cities experienced a more humid period in Ming (AD 1368–1644), with 26 records of anomalous rainfall events, which is the most among all dynasties [41], and records of dry springs starting to gush again in AD 1540 (Sichuan) [42] and AD 1769 (Hunan) [43]. Those records match the reconstructed groundwater level changes the ancient water wells revealed.

The other pattern observed in coastal cities (i.e., Yancheng, Fuzhou, and Guangzhou) exhibited a characteristic PGWL with a relatively high level during Song (AD 960–1279) and a sharp decline during Ming (AD 1368–1644). In Ming (AD 1368–1644), PGWL dropped to –9.60 m a.s.l. in Yancheng, –2.70 m a.s.l. in Suqian, and –0.06 m a.s.l. in Guangzhou, which were the lowest across all dynasties. This pattern also shares two common periods with the above pattern: high PGWL during Tang (AD 618–907) and low PGWL during Jin (AD 266–420). These periods can be demonstrated by some extreme events recorded in the *Chronology of Major Natural Disasters and Anomalies in Ancient China* [41]. During Song (AD 960–1279), there were more severe floods and rainfall events than droughts [44], suggesting a high water table. In contrast, some water wells and rivers dried up during Yuan and Ming (in AD 1240, 1247, 1458, and 1462 in Zhejiang and AD 1471 in Yangzhou), indicating a drop in the water table during those periods. Moreover, the pattern of groundwater changes in other cities (Nanjing, Suzhou, and Suqian) is not distinctive.

### PGWL fluctuation and climate variation

**Fig 6:**
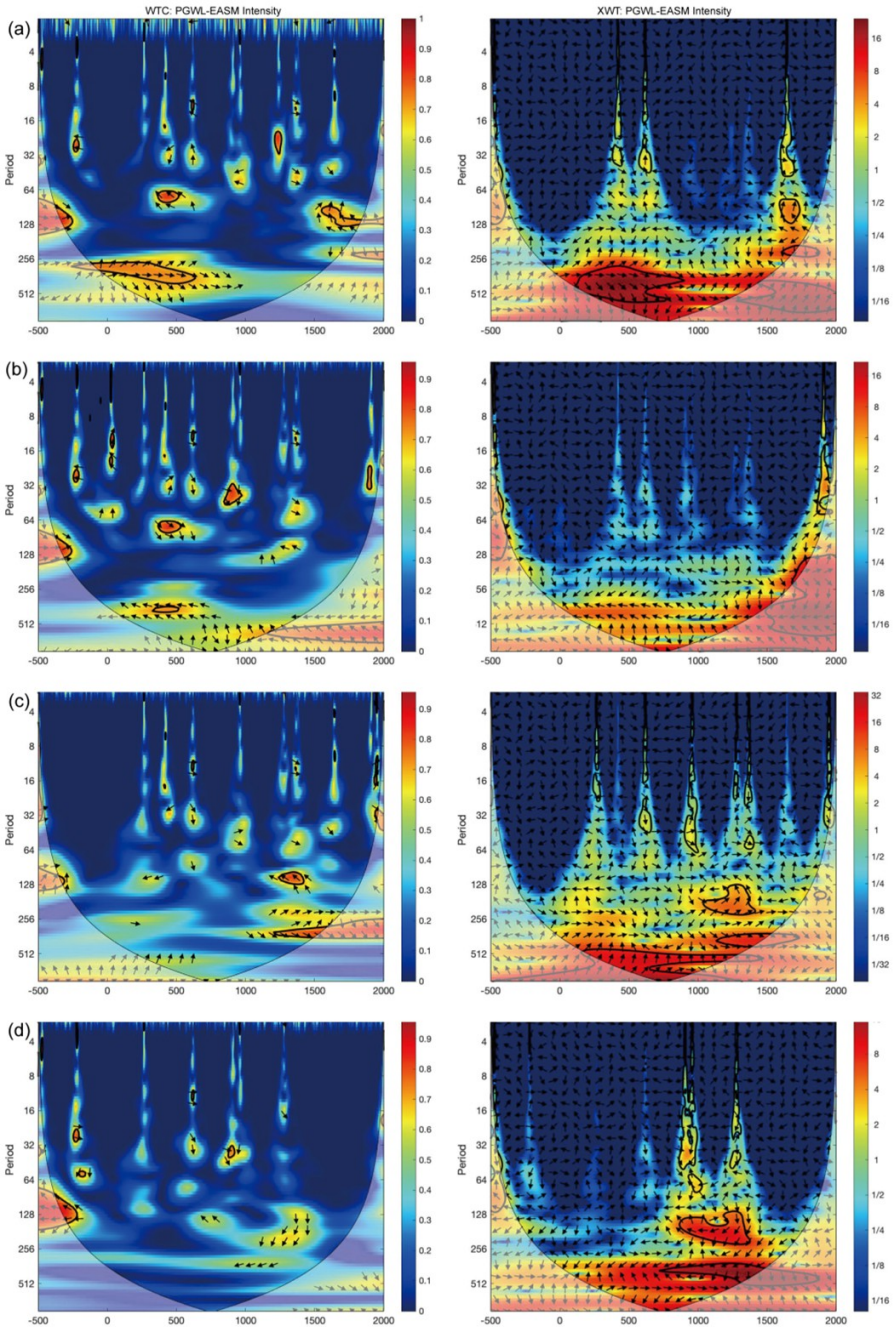
WTC and XWT of the EASM intensity [45] with the PGWL in (a) Chengdu, (b) Changsha, (c) Nanjing, and (d) Suzhou. The legend reveals the coherence value ranging from low (dark blue) to high (dark red). The 5% significance level against red noise is shown in the black contour. Edge effects of the region shaded outside the influence cone may distort coherence.

**Fig 7:**
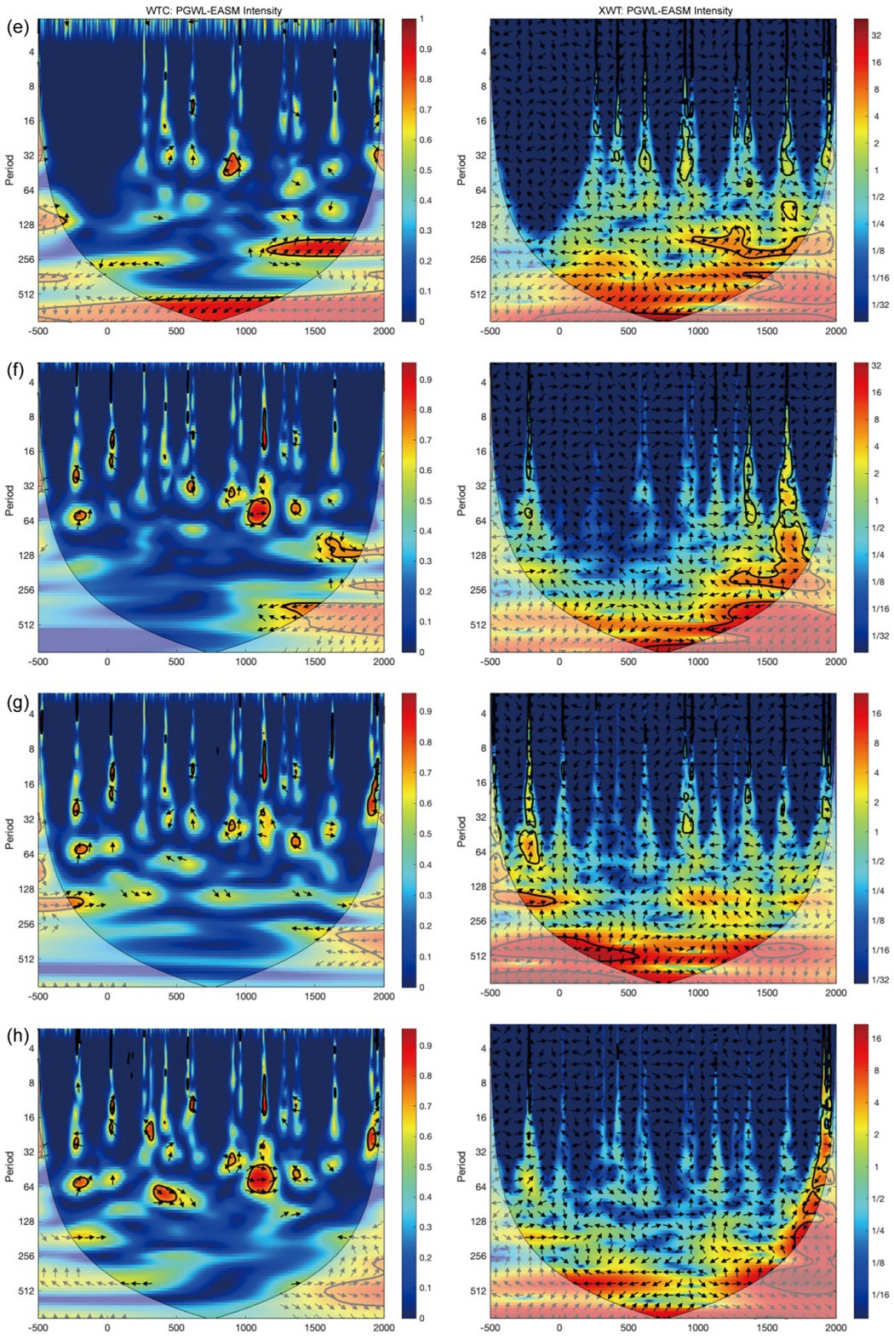
WTC and XWT of the EASM intensity [45] with the PGWL in (e) Suqian, (f) Yancheng, (g) Fuzhou, and (h) Guangzhou. The legend reveals the coherence value ranging from low (dark blue) to high (dark red). The 5% significance level against red noise is shown in the black contour. Edge effects of the region shaded outside the influence cone may distort coherence.

**Fig 8:**
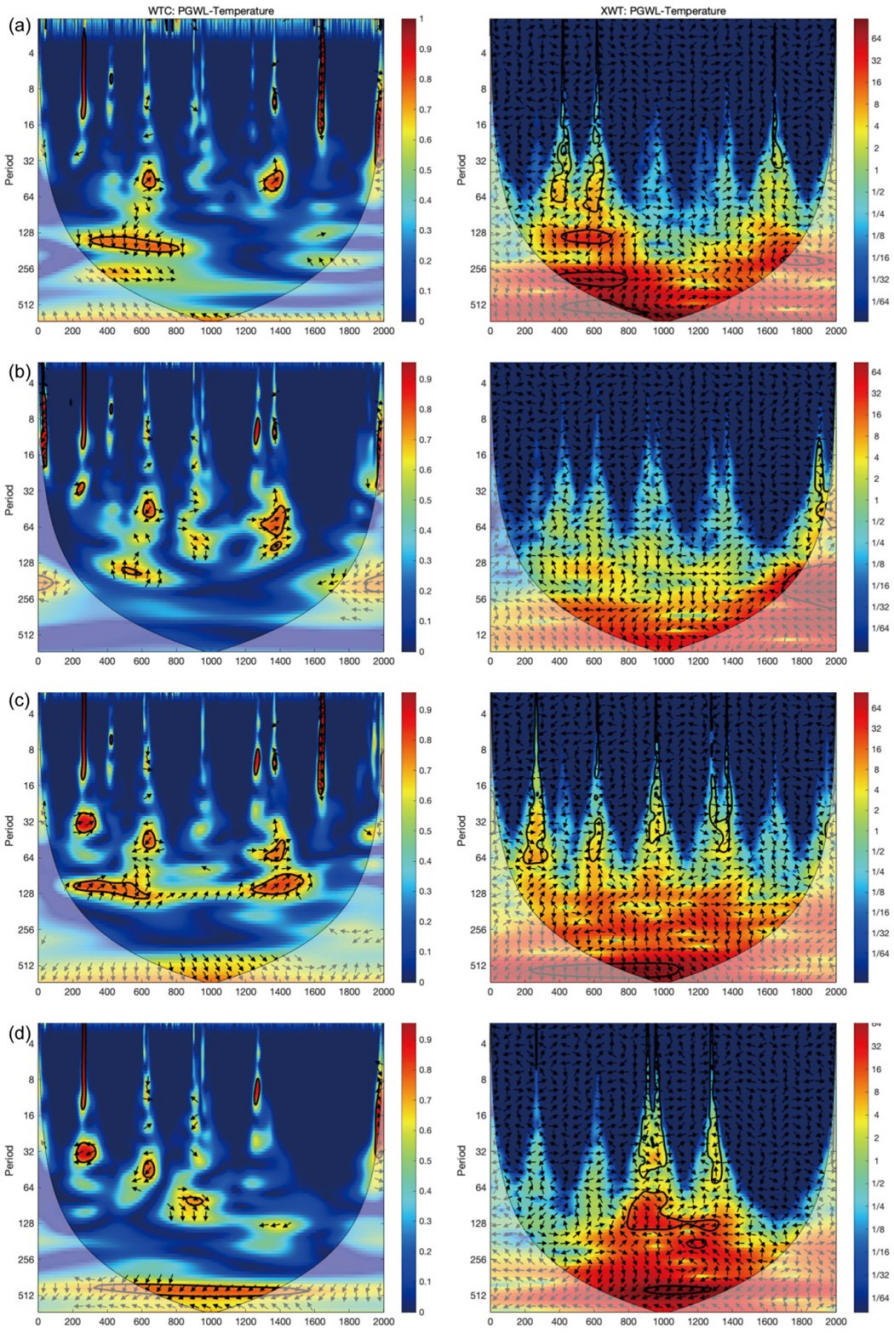
WTC and XWT of the east-central China temperature [46] with the PGWL in (a) Chengdu, (b) Changsha, (c) Nanjing, and (d) Suzhou. The legend reveals the coherence value ranging from low (dark blue) to high (dark red). The 5% significance level against red noise is shown in the black contour. Edge effects of the region shaded outside the influence cone may distort coherence.

**Fig 9:**
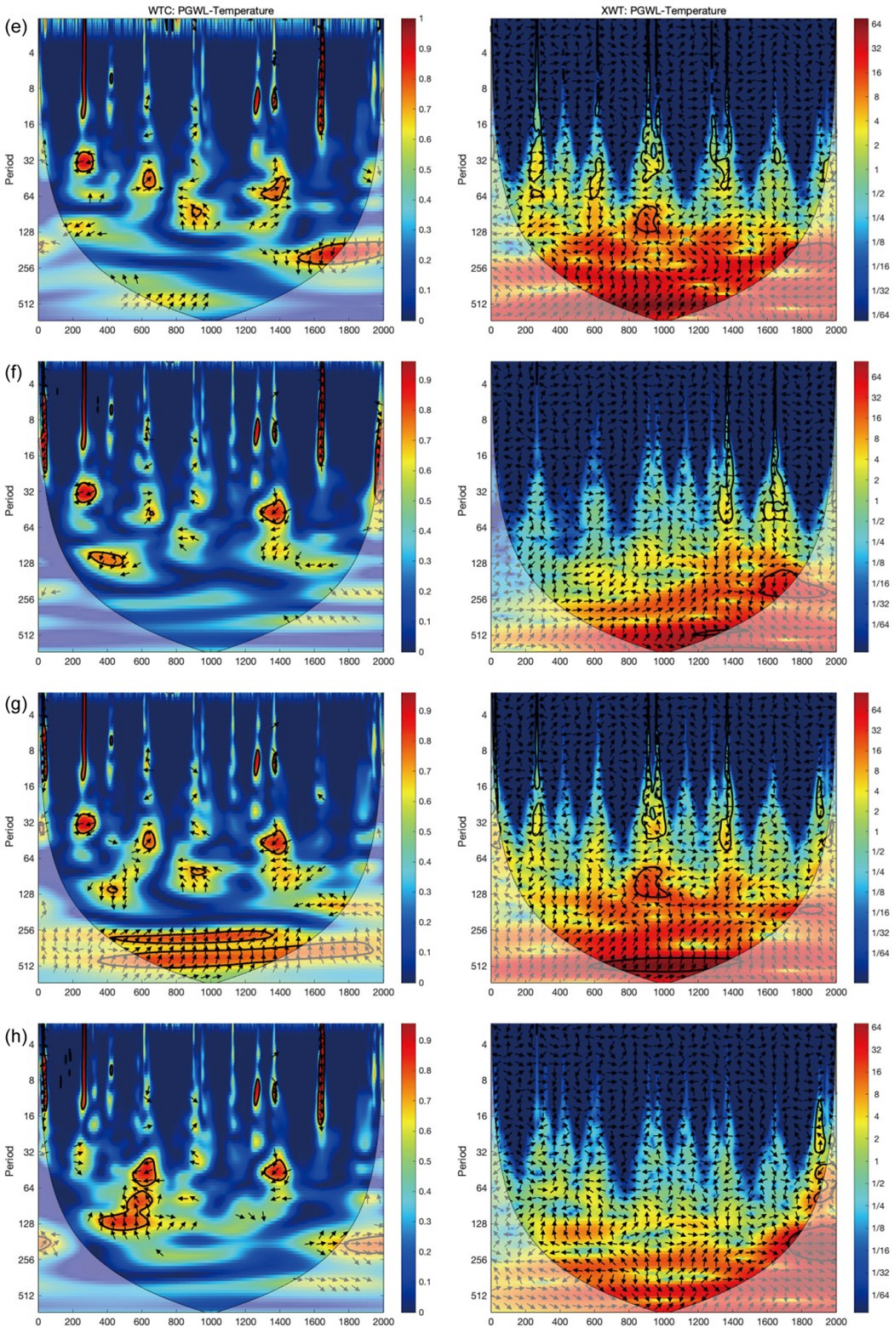
WTC and XWT of the east-central China temperature [46] with the PGWL in (e) Suqian, (f) Yancheng, (g) Fuzhou, and (h) Guangzhou. The legend reveals the coherence value ranging from low (dark blue) to high (dark red). The 5% significance level against red noise is shown in the black contour. Edge effects of the region shaded outside the influence cone may distort coherence.

To investigate the temporal and multi-scale correlation between groundwater level fluctuations and climate change, we conducted the XWT and WTC analyses on the dynastic average of PGWL using the time series of East Asian Summer Monsoon (EASM) intensity and temperature during the historical period, respectively (Figs 6–9).

The left column of Figs 6 and 7 shows the multi-scale synchrony of the PGWL fluctuations for eight cities with the EASM intensity reconstructed from oxygen isotope records of stalagmites in the Dongge Cave [45]. The figure shows that the PGWL in Chengdu and Changsha cohered with EASM at a 256–512 year periodicity between 500 BC and AD 2000. These two cities are the only ones whose PGWL cohered with EASM intensity over the entire time domain. In other cities, the coherence between PGWL and EASM only existed around AD 1000. For example, in Nanjing, coherence occurred in a 250–450 year cycle after AD 1000 and a 120–130 year cycle after AD 1200. In Suzhou, the coherence occurred in a 130–250 year cycle after AD 1000, while in Suqian, the coherence occurred in a 150–250 year cycle after AD 1200. In addition, there was coherence between PGWL and EASM in all cities after AD 1300 (although some of it failed the noise test at the 95% confidence level, which should be interpreted with caution). The cross-wavelet spectrum on the right-hand side of Figs 6 and 7 show the high-energy resonance period, basically the same as the periods mentioned above, where a strong correlation exists.

Figs 8 and 9 illustrate the coherence between PGWL and the east-central China temperature [46]. All cities exhibit a cycle of around 128–200 years during AD 200–700 and 1200–1600. For instance, Chengdu shows a coherence with a 128–210 year cycle during AD 200–820 and a 200–250 year cycle during AD 1450–1800. On the other hand, Nanjing and Fuzhou had a 100–160 year cycle during AD 200–1600 and 250–512 during AD 400–1400. Furthermore, most cities show a periodicity of 128–300 years after AD 1400, except for Nanjing, with a periodicity of 512 years, and Suzhou, with a periodicity of 450–512 years. Suqian, Yancheng, Fuzhou, and Guangzhou show coherence between PGWL and temperature at cycles ranging from 128 to 256 years. It is worth mentioning that although some of the coherence of long periodicity is in the shaded region and may be distorted by the edge effect, it still reflects the potential existence of long cycles. It can be inferred that the long cycles continued to exist in Suqian, Yancheng, and Fuzhou after AD 1400, while the short cycles may have changed to the long ones in Nanjing, Suzhou, and Guangzhou. The cross-wavelet spectrum in the right column indicates high coherence between the PGWL fluctuations and temperature variations during the abovementioned periods.

## Discussion

Different factors likely influenced the patterns of the PGWL variations in different places and periods. This section discusses human behaviors and climatic factors that are believed to have influenced PGWL at the centennial scale.

### Influence of anthropogenic factors on the PGWL variations

Although human activities such as irrigation, population growth, and building water facilities cannot be neglected during imperial periods, they are not considered to have a significant long-term impact on the variation of the PGWL. Irrigation use was considered a primary reason for the drop in groundwater levels during imperial times [47, 48]. However, the use of well water for irrigation and manufacturing was not widespread until Ming (AD 1368–1644) and Qing (AD 1644–1911) [3], and the shortage of agricultural water occurred more in North China than in the south. Our study region is located in South China. Abundant water sources from the Yangtze River and Huai River allowed ancient people to irrigate with surface water rather than groundwater, which the installation of farmland irrigation facilities can reflect. Contrary to numerous historical accounts of the use of well irrigation methods in the north [49], agricultural irrigation facilities like water-proofing polders and drainage were primarily used in South China to manage surface water resources rather than to exploit groundwater [50, 51].

Additionally, historical household water use is unlikely to have contributed to the changes in groundwater levels in our study area for the following reasons: First, people in southern China did not have to rely solely on groundwater supplies. While well water was typically used for drinking and cooking, river water was more frequently used for washing and irrigation [52]. Second, even in modern times, the drop in the groundwater table is not primarily caused by intensified domestic water use brought on by population growth. The responsibility for the groundwater table drop should lie with the use of groundwater for industrial production. This is because groundwater used for industrial production is extracted in large quantities from the pressurized water layer, which can lead to major land subsidence incidents, as evident in some cities in eastern China [53, 54]. Ancient wells were generally only able to be drilled to the phreatic layer due to drilling technology limitations. Together with the lack of industry at the time, it implied that ancient wells were unlikely to alter the groundwater table.

Moreover, very few large-scale hydraulic engineering projects (e.g., dams) that would raise river levels are found in the study areas. However, the Beijing-Hangzhou Grand Canal, which passes through Nanjing and Suzhou, drew our attention. Canals were supposed to use rivers and lakes to function, meaning many rivers were cut off and shrank, causing groundwater to replenish rivers in reverse [49]. This might explain why the PGWL in Nanjing, Suzhou, and Suqian declined even during the humid Tang (AD 618–907). Nevertheless, the canal would have quickly found a new equilibrium with the nearby river systems, so this effect would have had little impact in causing groundwater variation over a long temporal scale. The analysis of contemporary groundwater demonstrates further that the canal has not impacted the strength of groundwater-monsoon resonance frequencies [56]. Even though anthropogenic activities have significantly decreased groundwater flow, climate change plays a major role in affecting the evolution of hydrological drought on longer time scales [57]. We infer from the aforementioned arguments that PGWL in Southern China was more closely related to climate change than human activities during the imperial periods.

### Monsoon precipitation and the PGWL variation

**Fig 10:**
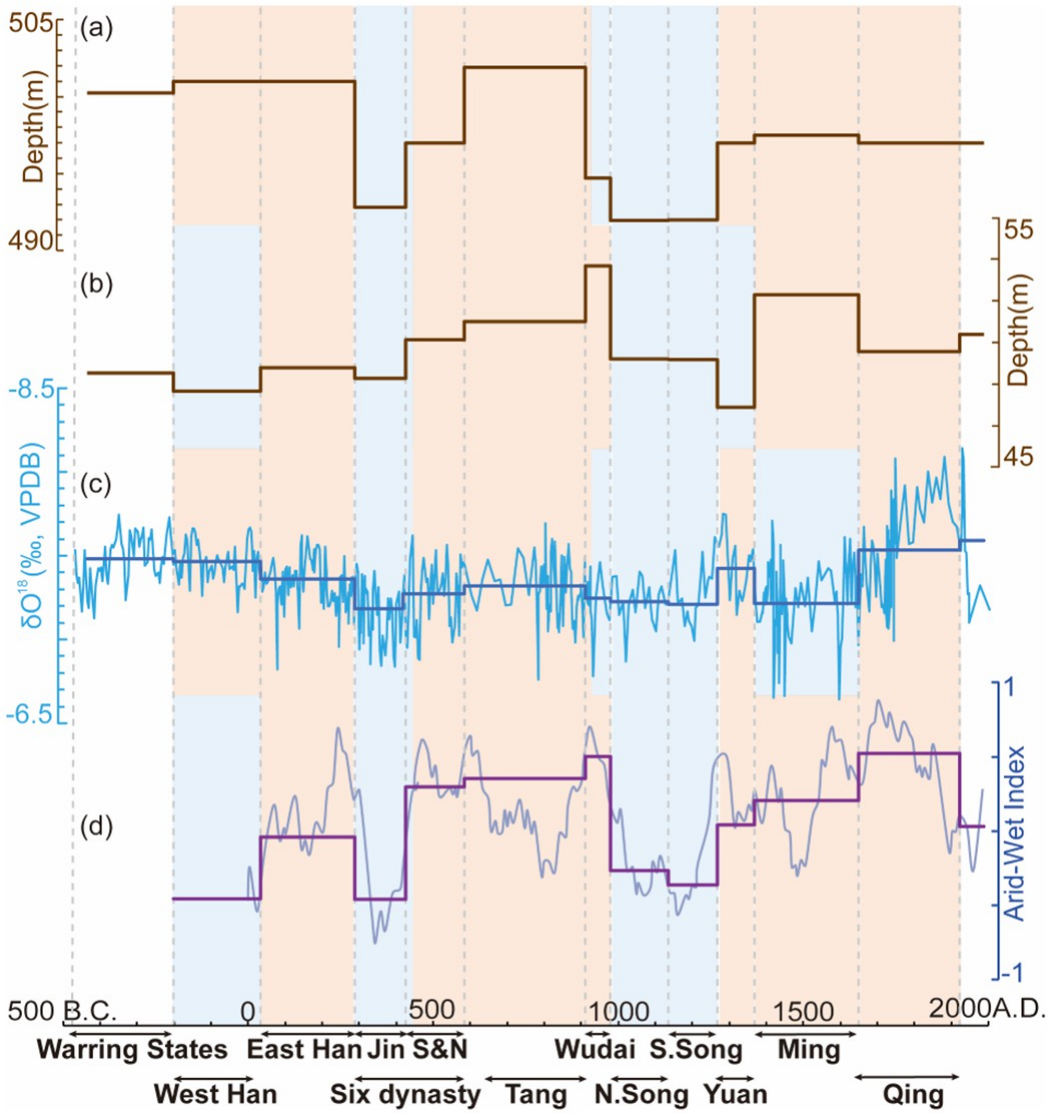
Comparison of the PGWL in Southern China’s inland monsoon region (Chengdu and Changsha), EASM, and Wet/dry index in the Jiangnan region. (a) PGWL in Chengdu; (b) PGWL in Changsha; (c) δ^18^O records derived from the stalagmites in the Dongge Cave [45]; (d) Wet/dry Index in the Jiangnan Region reconstructed from historical documents [52].

Precipitation directly affects PGWL by increasing or decreasing groundwater recharge, so changes in precipitation can be seen as the cause of PGWL fluctuations. Our sampled areas are all in the EASM region, where the monsoon mainly brings precipitation to these cities. Based on our wavelet coherence results (Figs 6 and 7), we found that EASM intensity primarily affects Chengdu and Changsha on a long-term periodicity (>256 years) and shows a similar pattern in fluctuation trends (Fig 10), probably because Changsha and Chengdu are on the same front of the climatic zone [50] (i.e., inland monsoon region). Their precipitation is mainly derived from Indian Ocean water vapor, so precipitation variation indicated by PGWL and monsoon intensity is broadly consistent [45, 51]. However, this relationship shows inconsistency in the Ming dynasty: monsoon intensity is weak, but precipitation is intense, while precipitation is more consistent with the humidity reconstructed by historical documents [52]. This finding suggests that the historical record and the rise of PGWL accurately reflect the increased precipitation in Ming (AD 1368–1644), even as the monsoon intensity weakened.

This is because the difference in the atmospheric response between the Asian continent and the adjacent ocean largely determines the strength of the Asian monsoon. The weak circulation of EASM during Ming (AD 1368–1644) allowed the EASM rain belts to remain in the south, resulting in increased rainfall in the south and decreased rainfall in the north [53]. In contrast, the monsoon intermittently influenced other regions (Figs 6 and 7), with fluctuations in intensity after AD 1200. The monsoon intensified significantly after AD 1200–1400 and 1700, with its influence extending eastwards. During these periods of monsoon intensification, some regions were significantly disturbed and thus do not show a typical pattern of groundwater change, such as Nanjing and Suzhou. The pattern in Suqian, on the other hand, is more similar to that of other coastal cities, probably because the Pacific Ocean more significantly influenced it. Hence, the impact of the EASM on Suqian is limited, even though it was strengthened. This difference is also found most notably in the Ming dynasty.

### Temperature and the PGWL variation

**Fig 11:**
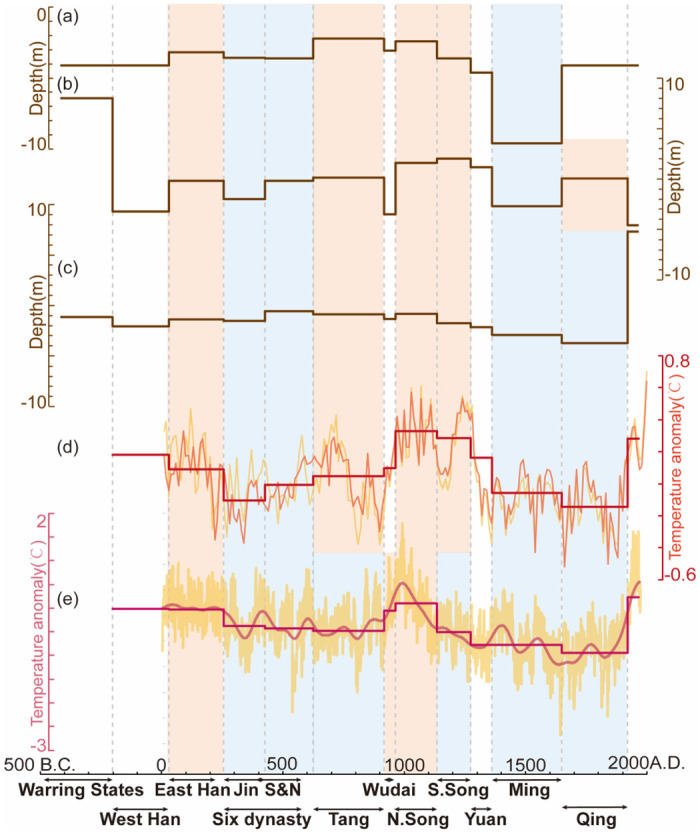
Comparisons of the PGWL in Southern China’s coastal region (Yancheng, Fuzhou, and Guangzhou) and temperature in eastern-central China and extratropical Northern Hemisphere. (a) PGWL in Yancheng; (b) PGWL in Fuzhou; (c) PGWL in Guangzhou; (d) Winter half-year temperature changes in eastern-central China [46]; (e) Extra-tropical Northern Hemisphere temperature [62].

Based on the XWT and WTC results, the temperature has a long-term effect on the PGWL in Nanjing, Suzhou, and Fuzhou. In addition, it correlates with Guangzhou at 32–130 year cycles between AD 200–1400 and 130–256 year cycles after AD 1400. As for Chengdu and Changsha (i.e., inland monsoon region), the effects of temperature are intermittent and primarily occur in short cycles. Fig 11 shows that the pattern of PGWL fluctuations in Yancheng, Fuzhou, and Guangzhou (i.e., coastal region) is coherent with the temperature in both eastern-central China and the extra-tropical North Hemisphere (NH). Therefore, it can be deduced that temperature mainly affects the PGWL fluctuations in the coastal region on a long-term scale. Specifically, previous studies found a significant correlation between high sea level and temperature [54, 55], and some models based on temperature simulations of past sea level changes also showed this correlation [56, 57]. This is because an increase or decrease in temperature can lead to the melting or growth of glaciers [58–60], resulting in the rise or fall of sea level [61].

The increase in records of seawall construction and tidal flooding, as well as the removal and combination of counties in the Yangtze estuary area and the increase of flooding in the northern Jiangsu Plain and the Taihu Plain area, indicate a period of high sea level during Tang (AD 618–907) and Song (AD 960–1279) [63]. Conversely, the rapid rise of beach silting in the Yangtze and Yellow River estuary area after the Ming Wanli (AD 1572) reflects a drop in sea level [63]. These low (high) sea surface periods correspond to cold (warm) periods in the last two millennia [46, 52, 64], which also correlate with low (high) groundwater table.

Moreover, the short-term temperature-PGWL dynamics are evident in various cities in two distinct periods: AD 500–1000 and 1200–1600. These periods correspond exactly to the Medieval Warm Period (MWP) [65] and Little Ice Age (LIA) [66], which are known to have strong hydro-climatic anomalies [67], including alternating mega-floods and mega-droughts. For instance, the Yangtze River basin experienced extreme floods in AD 1153 and 1368 [68], and there are 17 records of heavy rains and floods in the southeastern coastal region during Ming (AD 1368–1644) [41]. The worst drought also occurred during Ming (AD 1368–1644), triggering 71 famines [41]. The dry climate of East China is evident in climate proxies from sedimentary strata. Examples include peat records from Jinchuan in Jilin [62] and Xiyaohu in Jiangxi [63]. After AD 1400, the PGWL of all cities showed a cyclical correlation with temperature. The climatic variability of LIA had an impact on PGWL in both eastern and western China.

We also employed XWT and WTC to compare PGWL with the extra-tropic NH temperature (S1 and S2 Figs). We found that both the extra-tropic NH and eastern-central China temperatures have similar long-term impacts on the PGWL in those coastal cities (Yancheng, Fuzhou, and Guangzhou). However, when the eastern-central China temperature data were used in XWT and WTC analyses, it can be seen that the temperature affects PGWL in shorter cycles in the inland monsoon region (Chengdu and Changsha) (Fig 8), while such a phenomenon could not be found when the extra-tropic NH temperature data were used (S1 Fig). Those shorter cycles may be attributed to the short-term changes of evapotranspiration brought about by regional temperature change. Furthermore, the temporal difference in the temperature effect on PGWL between inland monsoon and coastal regions reveals the discrepancy in the way that temperature affects groundwater across Southern China (i.e., the longer cycle of the temperature influence on the PGWL in the coastal region is accomplished by the temperature-induced sea level changes at the hemispheric scale, while the shorter cycle of the temperature influence on the PGWL in the inland monsoon region is caused by the temperature-induced evapotranspiration changes at the regional scale.

## Conclusions

The PGWL, reconstructed from 511 ancient wells in eight cities in Southern China, reveals two distinct fluctuation patterns. One pattern, represented by Chengdu and Changsha in the inland monsoon region, shows a high PGWL during Tang (AD 618–907) and Ming (AD 1368–1644) and a low PGWL during Jin (AD 266–420) and Southern Song (AD 1127–1279). The other pattern in Yancheng, Fuzhou, and Guangzhou in the coastal region shows a high PGWL in Tang (AD 618–907) and Song (AD 960–1279) and a low PGWL in Jin (AD 266–420) and Ming (AD 1368–1644). The XWT and WTC analyses of the multi-scale correlation between climate variables and PGWL show that monsoon intensity and temperature affect PGWL at the inter-centennial scale. However, there are periodic differences across time and locations. Monsoon intensity mainly affects PGWL in Chengdu and Changsha in the inland monsoon region at the inter-centennial scale (256–512 year cycle). At the same time, Nanjing and Suzhou show a relatively short-period correlation with the monsoon when the monsoon strengthens. Temperature affects long-term PGWL in coastal areas via sea-level changes. Meanwhile, the PGWL in the inland monsoon region is also intermittently affected by temperature during climatic anomalies (MWP and LIA). Ancient wells, the archaeological materials used in this study, are directly associated with the hydrological environment. They demonstrate their potential use in hydro-climatic studies, as they can help us comprehend the history and driving mechanisms of PGWL.

## Supporting information

S1 File Data and reconstructed time series of PGWL in the eight cities covered in this study. (XLSX)

S1 Fig WTC and XWT of the extra-tropic NH temperature [62] with the PGWL in (a) Chengdu, (b) Changsha, (c) Nanjing, and (d) Suzhou. (TIF)

S2 Fig WTC and XWT of the extra-tropic NH temperature [62] with the PGWL in (e) Suqian, (f) Yancheng, (g) Fuzhou, and (h) Guangzhou. (TIF)
