# Supplementary figures and images for: Changes in paleo-groundwater levels revealed by water wells and their relationship with climate variations in imperial Southern China
Source: PLoS One. 2023 Oct 25;18(10):e0292662. doi: 10.1371/journal.pone.0292662 (PMC10599520; doi:10.1371/journal.pone.0292662)

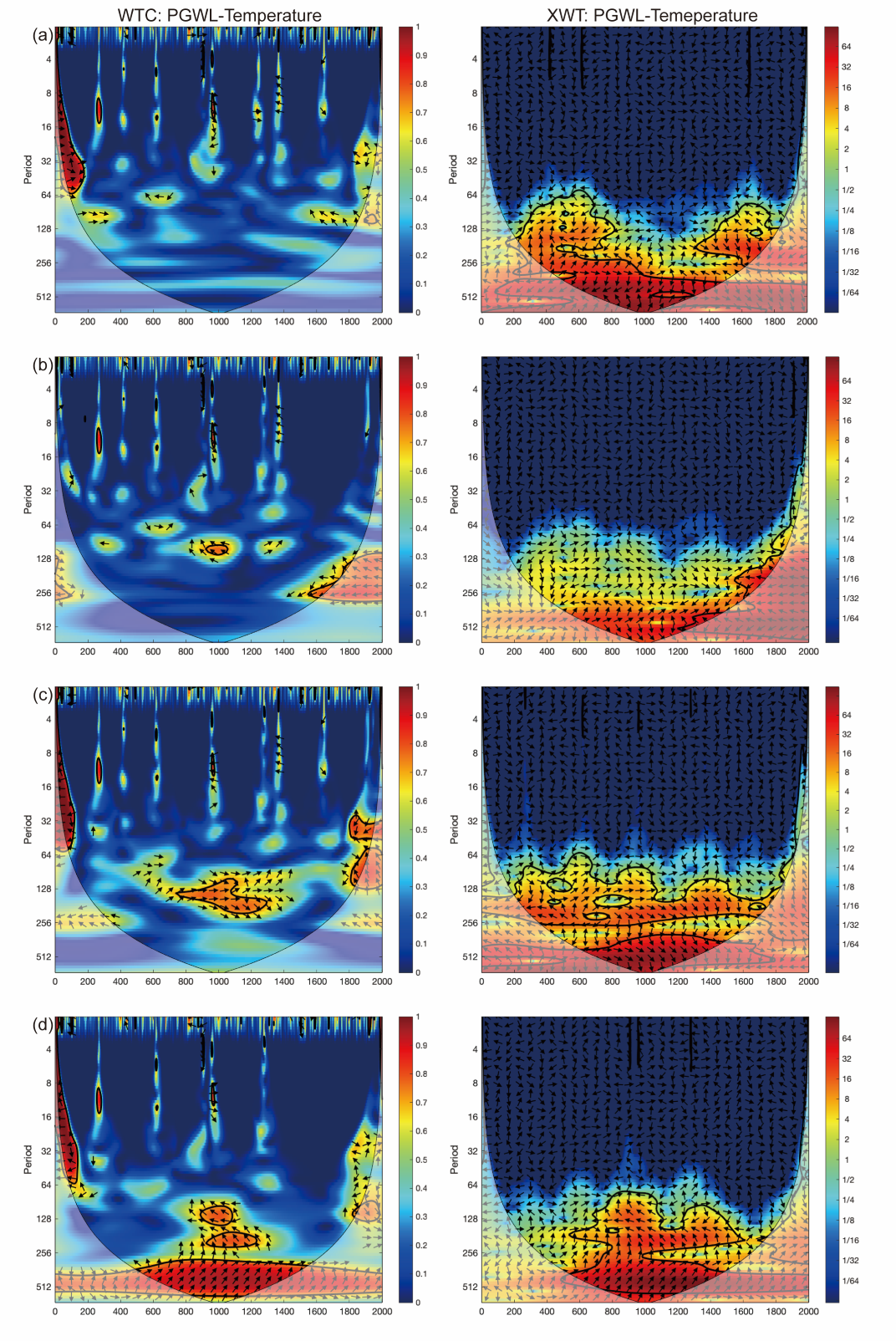

Supplement: S1 Fig — (TIF) [file pone.0292662.s002.tif]

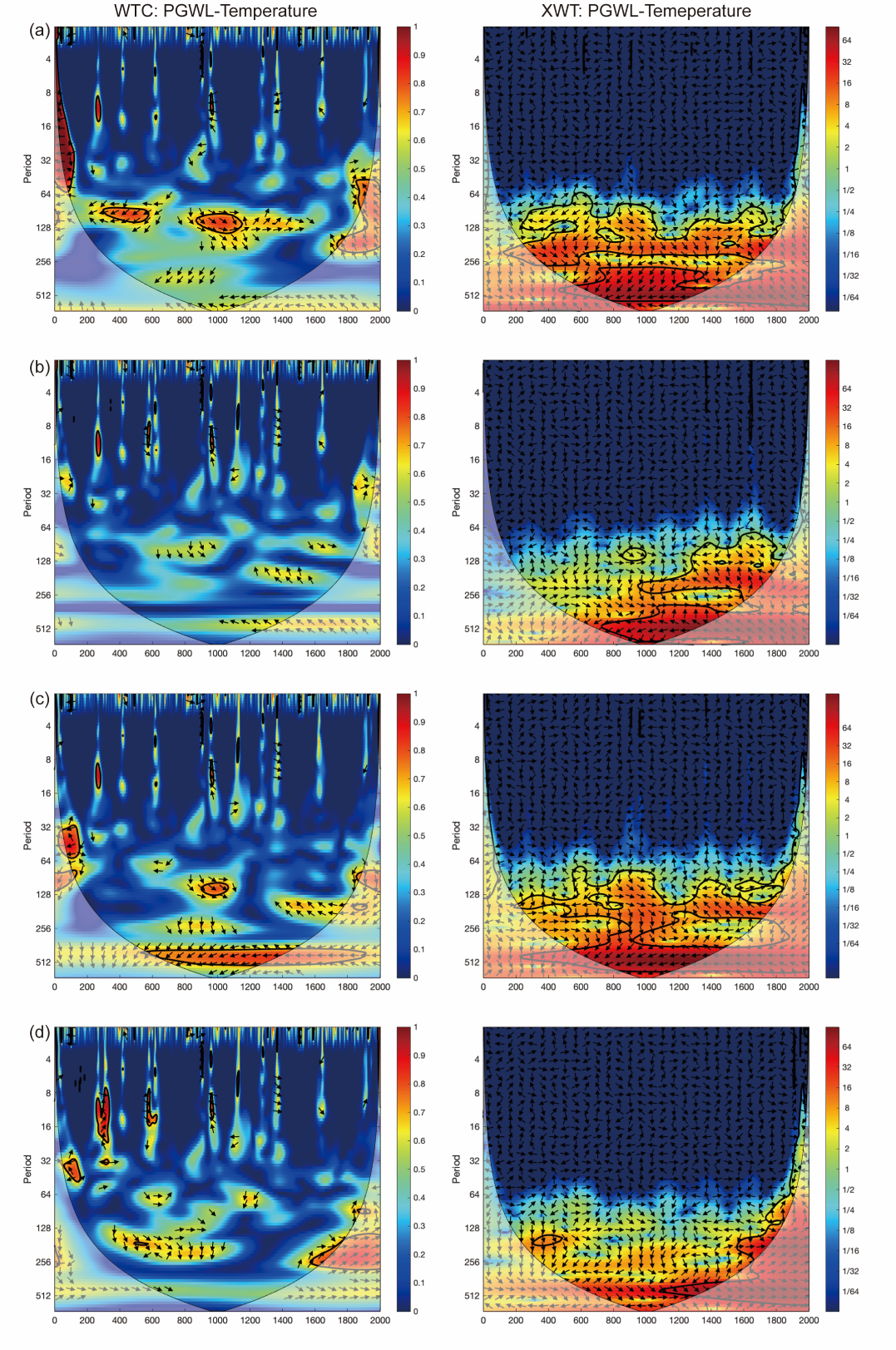

Supplement: S2 Fig — (TIF) [file pone.0292662.s003.tif]
